# The causal model of health literacy and health behavior for obesity prevention among primary school students in Bangkok, Thailand

**DOI:** 10.12688/f1000research.26249.2

**Published:** 2021-12-21

**Authors:** Ladaporn Thongsong, Wanida Neranon

**Affiliations:** 1 Department of Pediatric Nursing, Kuakarun Faculty of Nursing, Navamindradhiraj University, Bangkok, 10300, Thailand

**Keywords:** Causal Model, Health Literacy, Health Behavior, Obesity Prevention, Primary school students

## Abstract

**Background: **The aim of the study was to develop a research instrument to study the levels of health literacy for obesity prevention (HLFOP), as well as health behavior for obesity prevention (HBFOP). In addition, we investigated the causal model between health literacy and health behavior for obesity prevention among primary school students in Bangkok, Thailand.

**Methods**: A cross-sectional study among 600 participants who were primary school students (aged 9-13 years) was conducted. The participants were selected from schools in all parts of Bangkok using multi-stage random sampling technique. The research instrument to assess HLFOP and HBFOP, constructed by the researchers, were utilized for data collection. Data were analyzed using descriptive statistics, exploratory and confirmatory factor analyses, and structural equation model through linear structural relationship.

**Results**: We found that HBFOP was directly influenced by heath literacy in the category of Critical Literacy with an effect size of 0.65 (p < 0.01), and was indirectly influenced in the category of Basic Literacy and Interactive Literacy through Critical Literacy with effect sizes of 0.46 and 0.58 (p<0.01), respectively. The model was consistent with the empirical data, with Chi-Square=13.68, df=7, p=0.05721, RMSEA (root mean square error of approximation)= 0.040,  SRMR (standardized root mean square residual)= 0.017 NFI (normal fit index)=0.99, GFI (goodness of fit index)=0.99, and AGFI (adjusted goodness of fit index)=0.97.

**Conclusions**: HLFOP was influential on HBFOP in primary school students in the Bangkok Metropolis. The categories that were particularly influential were: 1) Basic Literacy: accessing health information skills; 2) Interactive Literacy: communication skills; and 3) Critical Literacy: media literacy and self-management skills.

## Introduction

Obesity in children has become a challenging health problem, not only in high-income countries but also in low and middle-income countries, especially in urban areas ( World Health Organization (WHO), 2000). Medical evidence has recently indicated that obesity and overweight children are highly risky of acquiring various non-communicable diseases (NCDs), which can result in premature death and disability in adulthood. They can also cause serious conditions of physical and mental health in children ( Society of Pediatric Nutrition of Thailand, 2014; WHO, 2000). Causes of obesity and being overweight are often due to changes in the diet and physical activity patterns, environmental and societal changes associated with urban development, and lack of supportive policies from various authorities, such as health, urban planning, environment, food processing distribution, marketing and education. The World Health Organization (WHO) reported that the global prevalence of overweight and obesity among children and adolescents aged 5–19 years has dramatically risen from 4% in 1975 to 18% in 2016. The rise has occurred similarly among both boys and girls; in 2016 18% of girls and 19% of boys were overweight ( WHO, 2019).

The WHO has implemented Global Strategies on Diet, Physical Activity and Health in order to help reduce the rising level of NCDs due to the steady increase in obesity in children worldwide ( WHO, 2009a). This indicates that most countries in the world, including high-, mid- and low-income nations, are suffering from obesity, which is contributing to the increasing number of cases and deaths from NCDs.

In Thailand, overweight and obese school-age children remain high and the numbers of cases increase annually in most urban areas of all regions, especially in Bangkok Metropolis ( Health Systems Research, 2011; Health Systems Research, 2014; Ministry of Public Health, 2018. As compared to the global prevalence, there has been an upward trend in overweight and obese Thai children, especially those aged 6–14 years. Research has shown that the rate of obese and overweight boys has increased from 16.7% in 2009 to 26.1% in 2014, or by 56% in the five-year period higher than the global rate within 33 years ( Health Systems Research, 2014). In addition, Mohsuwan & Aekplakorn (2016) conducted a survey investigating the nutrition profile of Thai children between the ages of 1 and 14 nation-wide. The findings of the study revealed that overweight and obesity prevalence in urban areas was higher than in rural areas across all age groups. The study further showed that the prevalence of obesity in children aged 6–11 years living in Bangkok was the nation’s highest rate, followed by 12.9% of children aged 12–14, while 12.1% of the latter group was overweight. This suggests that Bangkok experiences significant childhood obesity and overweightness.

In 2016, 13.1% of children aged 6–12 years were overweight or obese. A study of health behavior of Thai children aged 12 years revealed that 69.9% of them consumed snacks, soft drinks and candies during their meals; 38.9% drunk soft drinks; and 26.6% drunk soft drink for more than three days a week. Moreover, snacks and candies were consumed by 76.9% and 58.6% of respondents, respectively ( Ministry of Public Health, 2016). Among those aged 10–14 years, only 27% did regular exercise, 72.3% used smartphones and computers (smartphones were most highly accessed in Bangkok compared with other areas), and 55.5% spent time playing computer games. However, children in Bangkok had insufficient intake of fruit and vegetables ( Health Systems Research, 2014; Mohsuwan, 2016). Consequently, overweight and obesity in Thai children is a crucial public health problem, which needs to be urgently solved. They not only affect children’s overall health condition, but also negatively impact on the country’s economic development ( Ministry of Public Health, Department of Health, 2016; Ministry of Public Health, Division of Non-Communicable Diseases, 2017). 

Heath literacy (HL) is a significant issue in Thailand that needs to be promoted to all age groups. The declaration of the Twelfth National Health Development Plan 2017–2021 stated that HL and health behavior (HB) were ultimate goals that needed to be increased and promoted. For instance, more appropriate exercise; consumption of more vegetables and fruits and less sweets, and fatty and salty food; purchasing quality healthy products; and reducing smoking and drinking ( Ministry of Public Health, Department of Health, 2016; Ministry of Public Health, National Health Development Plan, 2016). Significantly, HL has been defined as the cognitive and social skills that determine the motivation and ability of individuals to gain, access, understand and use information in promoting good health ( Nutbeam, 2000; Nutbeam, 2008; WHO, 2009a). Nutbeam divides HL into three levels, namely: basic/functional HL; interactive HL; and critical HL ( Nutbeam, 2000; Nutbeam, 2008). Furthermore, Manganello (2008) included media literacy as a fourth level of HL for adolescents, since they exploit more media and technology. The author emphasized that HL can be a factor that contributes to adolescents’ positive health outcomes in relation to HB, health costs and health service use. As seen in 17 studies conducted in adults and five studies in children, low HL was found significantly related to increased body mass index (BMI), and being overweight and obese. In cases of children and adolescents, the above relationship seems to be more consistent than the studies in adults ( Michou *et al*., 2018). Media literacy (ML) was positively related to total health promoting behavior scores, including prevention behaviors for cigarette smoking, nutritional and dieting habits, physical sedentary activity, safety and injury behaviors, and sexual behaviors ( Zamir *et al*., 2011). According to a systematic review, 13 studies stated that HL in basic skills and ML were significantly related to adolescents’ HB, but only two studies revealed the relationship between HL and HB of adolescents. Linking HL with HB of adolescents, as suggested by Fleary *et al*. (2018), is an interesting issue to be further investigated for a better understanding of the HL roles of adolescents. In addition, future research should examine the system of HL by developing an effective assessment tool for adolescents’ health behavior assessment ( Fleary *et al*., 2018; Michou *et al*., 2018).

As discussed above, Thai students in various regions still have low and moderate levels of HL and HB ( Behavioral Science Research Institute, 2014a; Department of Health Service Support, 2016). Currently, there is insufficient information concerning HL (specifically Interactive Literacy and Critical Literacy) in primary school students (aged 9–13 years) in Bangkok Metropolis. Therefore, this study aimed to develop a research instrument to study the levels of HL for obesity prevention (HLFOP), as well as HB for obesity prevention (HBFOP) and the causal model between HLFOP and HBFOP among primary school students. The research findings from this study could pave the way for curriculum development and HLFOP and HBFOP promotion among primary school students in both rural and non-rural areas.

## Methods

### Ethical considerations

This research project was approved by the Institutional Review Board, Kuakarun Faculty of Nursing, Navamindrathiraj University (approval number KFN-IRB 2017-07). The research was conducted under the Ethical Standard in Human Research of the National Policy and Guidelines for Human Research of Thailand. Information about the study was explained to the students, their parents and teachers prior to conducting the study, and written informed consent to participate in the study was signed and obtained from the students and their legal parents. As approved by the Research Fund Board of the University, each student participant received 100 baht cash incentive for their participation. Permission was sought from the students’ schools to conduct the study.

### Study design

This study was in two phases. In phase 1, HL and HB scales were developed to study the causal model between HLFOP on HBFOP. This research instrument was validated using pilot testing. In phase 2, a cross-sectional study was conducted using the research instrument created in phase 1 on 600 primary school students to assess the causal model between HLFOP and HBFOP in this population.

### Phase 1

***Development of study instrument.*** To understand HLFOP and HBFOP in Thai school-aged students, a questionnaire was systematically developed by the researchers. HLFOP and HBFOP factors were first synthesized by reviewing textbooks, research studies, articles and related documents to better understand the conceptual framework and principles for the construction of appropriate models, operational definitions and factor structures.

For the HLFOP, previous research that was applicable for the present study included that conducted by Nutbeam ( Nutbeam, 2008; Nutbeam, 2009) and Manganello ( Manganello, 2008; Manganello *et al.*, 2015) as well as some related previous studies in Thailand (e.g., Behavioral Science Research Institute, 2014a; Department of Health Service Support, 2016), while for HBFOP, especially applicable was the WHO’s conceptual framework on obesity prevention ( WHO, 2000; WHO, 2019), the “3Es, 2Ss” (Principles of NCD Prevention) from the Thai Ministry of Public Health ( Ministry of Public Health, 2016), and Principles of Medical Practice in Obesity Prevention and Treatment in Thai children by the Society of Pediatric Nutrition of Thailand (2014).

***Validation of study instrument.*** This research were development and validation of research tools according to guideline of Six-Stage Process for Structural Equation Modeling ( Hair *et al.*, 2010) and a number of previous studies especially, following the study of Thai people, the Behavioral Science Research Institute (2014b) and the Department of Health Service Support (2016). Content validity of the HLFOP and HBFOP research instrument was verified by five experts in the field of obesity, HL and HB in children and adolescents. All of the experts worked as child health specialists in Bangkok, including two child health and behavior specialists, one school health nurse, one children’s nutritionist, and one health education teacher of a primary school. After the expects checked the content validity of the research instrument, the researchers revised the instrument based on the experts’ feedback in terms of content, language use, and appropriate context representation for children aged 9–13 years in Bangkok. Index of consistence (IOC) was analyzed for criteria acceptability.

A pilot test was performed with 30 primary school students in Bangkok having the same characteristics as the target participants, which were students aged 9–13 years studying in Grades 4–6 (or Prathomsuksa 4–6) in schools under the Office of Bangkok Metropolitan Administration (BMA), with the ability to read, write, and communicate with normal movement, without congenital disease of metabolic syndrome and not being in weight control programs. Reliability was analyzed using Cronbach’s alpha. The revised version of the instrument was then used for data compilation. 

***Structure of study instrument.*** The questionnaire has three main sections. Section 1 surveys general demographic information of the participants. Section 2, consisting of 30 items, gauges the participants’ HLFOP in three dimensions, i.e. Basic Literacy (10 items), Interactive Literacy (10 items) and Critical Literacy (10 items). Section 3 assesses the participants’ HBFOP in three dimensions, including Eating Behaviours (12 items), Exercising Behaviours (6 items) and Emotional Control (6 items). The items in Sections 2 and 3 include both positive and negative statements (5 negative items for HLFOP and 10 negative items for HBFOP). The five-point Likert scale was used to investigate the HL and HB levels of the participants in terms of the frequencies they behaved according to each statement. The scale ranges from 5 = all the time, 4 = more often, 3 = sometimes, 2 = rarely, and 1= none. The score of negative questions were reversed. 

To interpret the scores of HLFOP and HBFOP of the participants, this study adopted the score interpretation criteria proposed by the study of Thai people, the Behavioral Science Research Institute (2014b) and the Department of Health Service Support (2016), the rules for the interpretation of the HLFOP and HBFOP scores are as follows: less than <60% indicating low level, 60–79.99% indicating moderate level, 80–100% indicating high level.

Following the study of Thai people, the Behavioral Science Research Institute (2014b) and the Department of Health Service Support (2016), the rules for the interpretation of the HLFOP and HBFOP scores are as follows: less than <60% indicating low level, 60–79.99% indicating moderate level, 80–100% indicating high level.

### Phase 2

***Participants.*** To determine an appropriate sample size to confirm a causal model, the sample size-to-parameters of 20:1 proposed by Hair *et al*. (2010) was adopted. Since there were 30 HLFOP parameters, the sample size of the present study was 600 participants.

**Figure 1.**
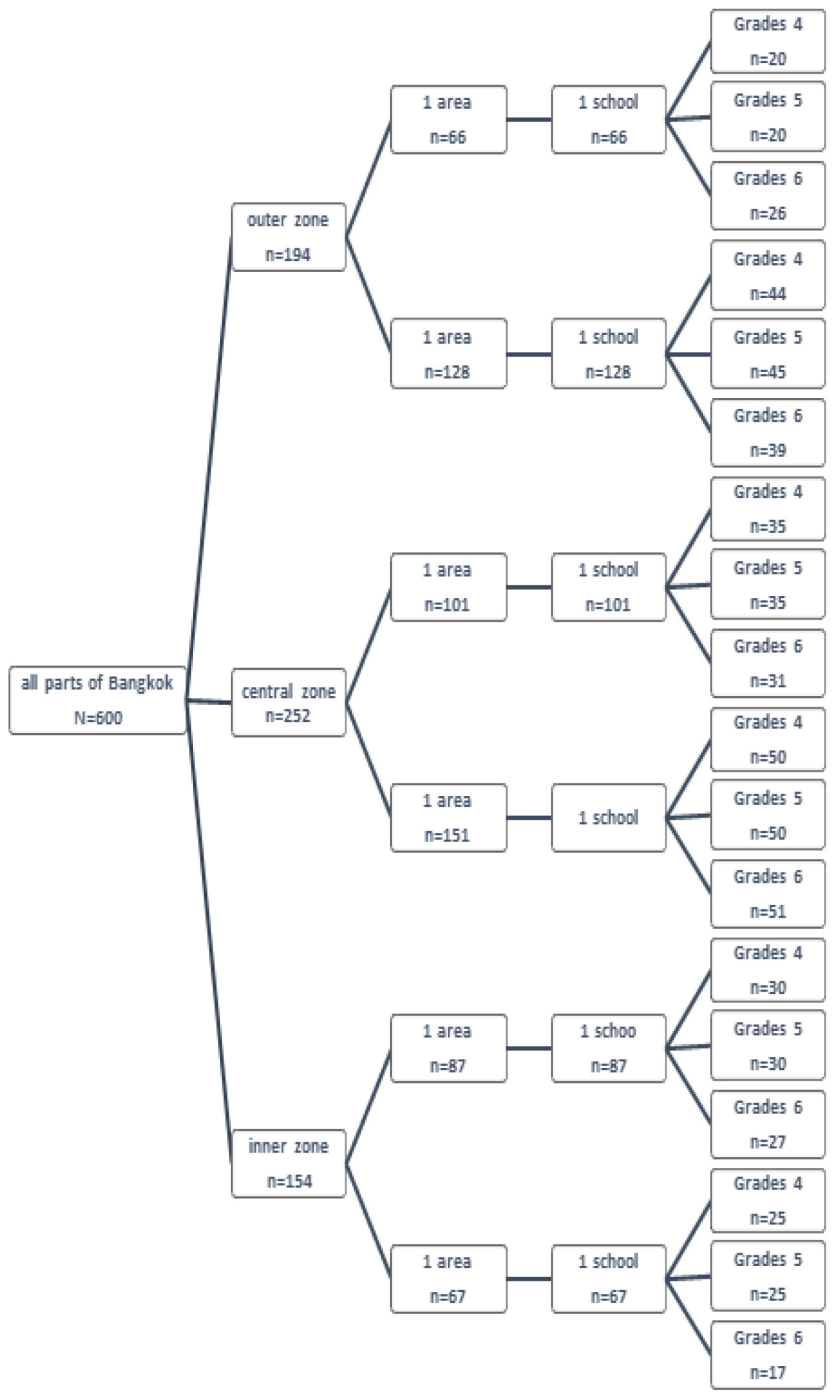
Chart for sampling.

The 600 students were selected using multi-stage random sampling from all parts of Bangkok. For more convenient selection for research participants, the researchers categorized all parts of Bangkok, generally comprised of 50 areas in total, into three main area categories, i.e. outer, central, and inner zones. Simple random sampling was then used to select two areas from each zone, contributing to the total of six areas, and one school in each area was further selected. Lastly, stratified random sampling was adopted to sample the target student participants from the three categorized zones, resulting in 194 students from the outer zone, 252 students from the central zone, and 154 students from the inner zone. ( Figure 1)

*Inclusion criteria:* primary school students aged 9–13 years studying in Grades 4–6 (or Prathomsuksa 4–6) in schools under the Office of Bangkok Metropolitan Administration (BMA), with the ability to read, write, and communicate with normal movement, without congenital disease of metabolic syndrome and not being in weight control programs.

***Data collection***. After obtaining consent from the students and their legal parents, a group of trained research assistants coordinated with the teacher coordinators of the six chosen schools. Then the research assistants went to the participants’ schools to collect data. Before starting compiling data, the research assistants gave self-introduction, expressed the purpose of the research clearly, and clarified the data collection details (e.g., length of time, rating description) to each of the participants. After having received and understood all the research-related information, each participant was given an untimed questionnaire to complete, taking normally 25–30 minutes to respond to the given questionnaire. The research assistants then double-checked each returned questionnaire to avoid blanks or incomplete responses.

### Statistical analysis

Descriptive statistics were utilized for data analysis of demographic information, and responses to the HLFOP and HBFOP instrument. Reliability of the instrument was analysed using Cronbach’s alpha, and exploratory factor analysis (EFA) and confirmatory factor analysis (CFA) were utilized to analyse construct validity. Structural equation model (SEM) through linear structural relationship was employed to analyze a causal model of HLFOP on HBFOP using LISREL 8.80.

## Results

### Development of the research instrument

**Figure 2.**
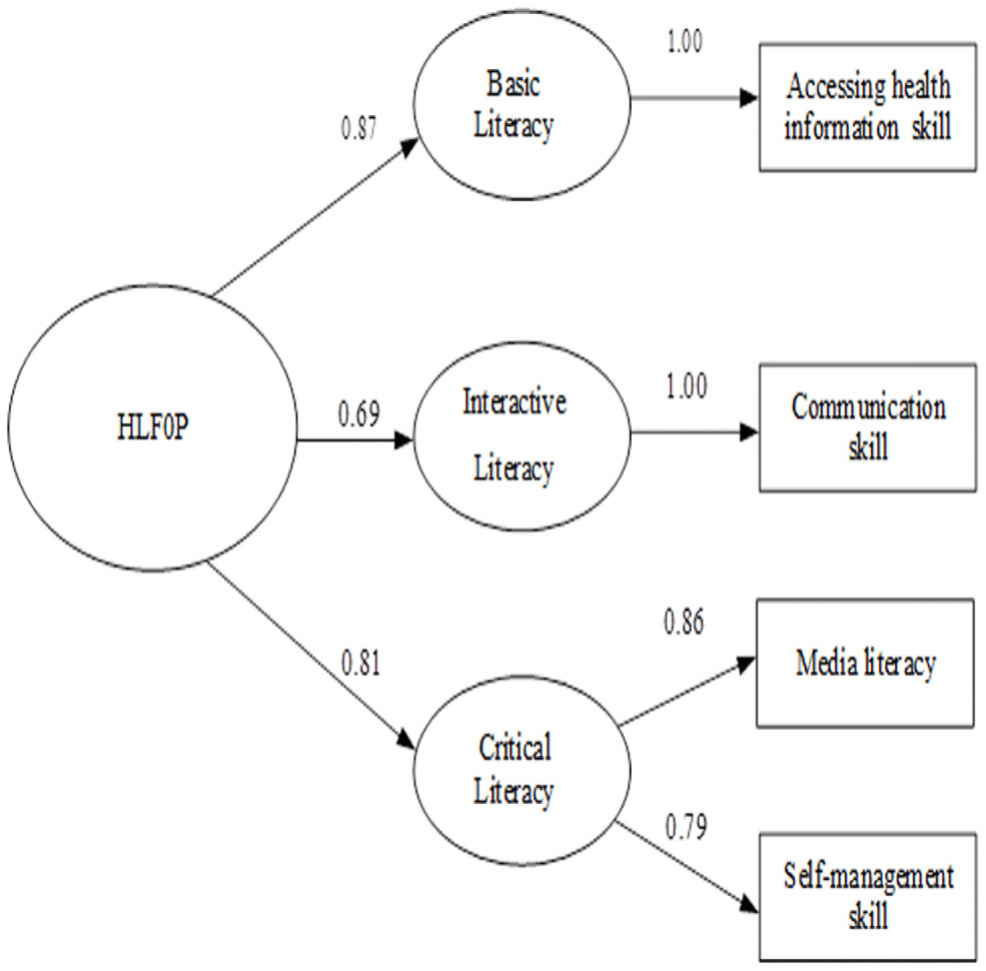
Confirmatory factor analysis of health literacy for obesity prevention among primary school students in Bangkok, Thailand. Chi-Square = 0.96, df = 1, P-value = 0.33, RMSEA = 0.000, SRMR = 0.0058, NFI = 1, GFI = 1 and AGFI = 0.99.

For section two (HLFOP), the construct validity of 18 items (out of 30) were approved by CFA. Three categories were classified: Basic Literacy (4 items on accessing health information skills); Interactive Literacy (5 items on communication skills); and Critical Literacy (4 items on ML and 5 items on self-management skills). Cronbach’s alpha for each category was 0.87, 0.78, 0.84, 0.76 and 0.81, respectively. Item total correlation coefficient was between 0.3–0.75. KMO = 0.928, Bartlett's Test = 3737.4 (p<0.000). Factor loading was between 0.69–0.87. The CFA was consistent with the empirical data (Chi-Square = 0.96, df =1, P-value = 0.33, RMSEA = 0.000, Standardized RMR = 0.0058, NFI = 1, Goodness of Fit Index (GFI) = 1, Adjusted Goodness of Fit Index (AGFI) = 0.99; Figure 2). 

**Figure 3.**
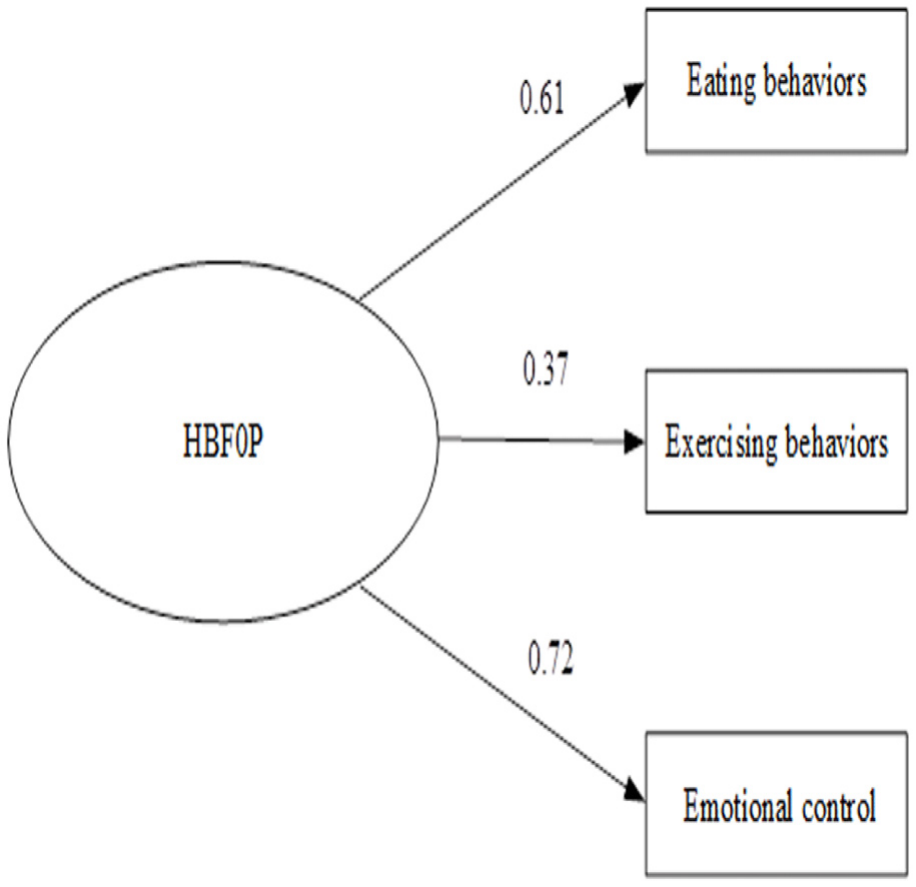
Confirmatory factor analysis of health behavior for obesity prevention among primary school students in Bangkok, Thailand. Chi-square = 0.00, df = 1, p-value = 0.977, RMSEA = 0.00.

For section three (HBFOP), the construct validity of 20 items (out of 24) were approved by CFA. Three categories were classified: Eating Behaviours (12 items); Exercising Behaviours (4 items); and Emotional Control (4 items). Overall Cronbach’s alpha was 0.73. Item total correlation coefficient was between 0.3–0.68. KMO = 0.863, Bartlett’s’ Test = 3412.40 (p<0.000). Factor loading was between 0.37–0.72. The CFA was found consistent with the empirical data (Chi-square = 0.00, df = 1, p-value = 0.977, RMSEA = 0.00; Figure 3). 

### Demographic data of the sample

A total of 600 students completed the study instrument, 246 boys (41%) and 354 girls (59%). In terms of school levels, 204 (34%) were from Prathomsuksa 4, 205 (34.2%) in Prathomsuksa 5, and 191 (31.8%) in Prathomsuksa 6. Their average age was 11.10 years (SD=0.94, Max = 13.3, Min = 9.00), with GPA= 3.39 (SD=0.51). Their average weight was 41.73 kgs (SD=13.5, Max =96, Min =20), and average height was 145.77cm (SD=9.8, Max = 176, Min =120). Most of the students (81%) resided with their parents, and 19% with relatives and other persons; 39.5% resided in rental houses and 23.8% in their own houses; 13.5% resided in flats or condominium, 6.7% in townhouses and 5% detached houses. Their parents’ marital status was as follows: 55.7% married or lived together, and 26% widowed, divorced or separated.

### Study the levels of HLFOP and HBFOP

The total means from students for HLFOP were at low level ( $\bar{X}$ = 45.76, SD = 12.77), whereas HBFOP were at moderate level ( $\bar{X}$ = 62.72, SD = 9.17). For HLFOP, 71.5% of students were at low level, 26.5% were at moderate level, and 2% were at high level. For HBFOP, 57.66% of students were at moderate level, while 39.1% were at low level at 39.17%, and 3.17% were at high level ( Table 1). 

**Table 1.** The levels of health literacy for obesity prevention (HLFOP) and health behavior for obesity prevention (HBFOP) for school aged children in Thailand.

Level interpretation	HLFOP			HBFOP		
	Range of total scores	N	%	Range of total scores	N	%
High	72–90	12	2	81–95	19	3.17
Moderate	54–71	159	26.5	60–79	346	57.66
Low	18–53	429	71.5	26–59	235	39.17
Total	18–90	600	100	20–100	600	100

### The causal model

**Figure 4.**
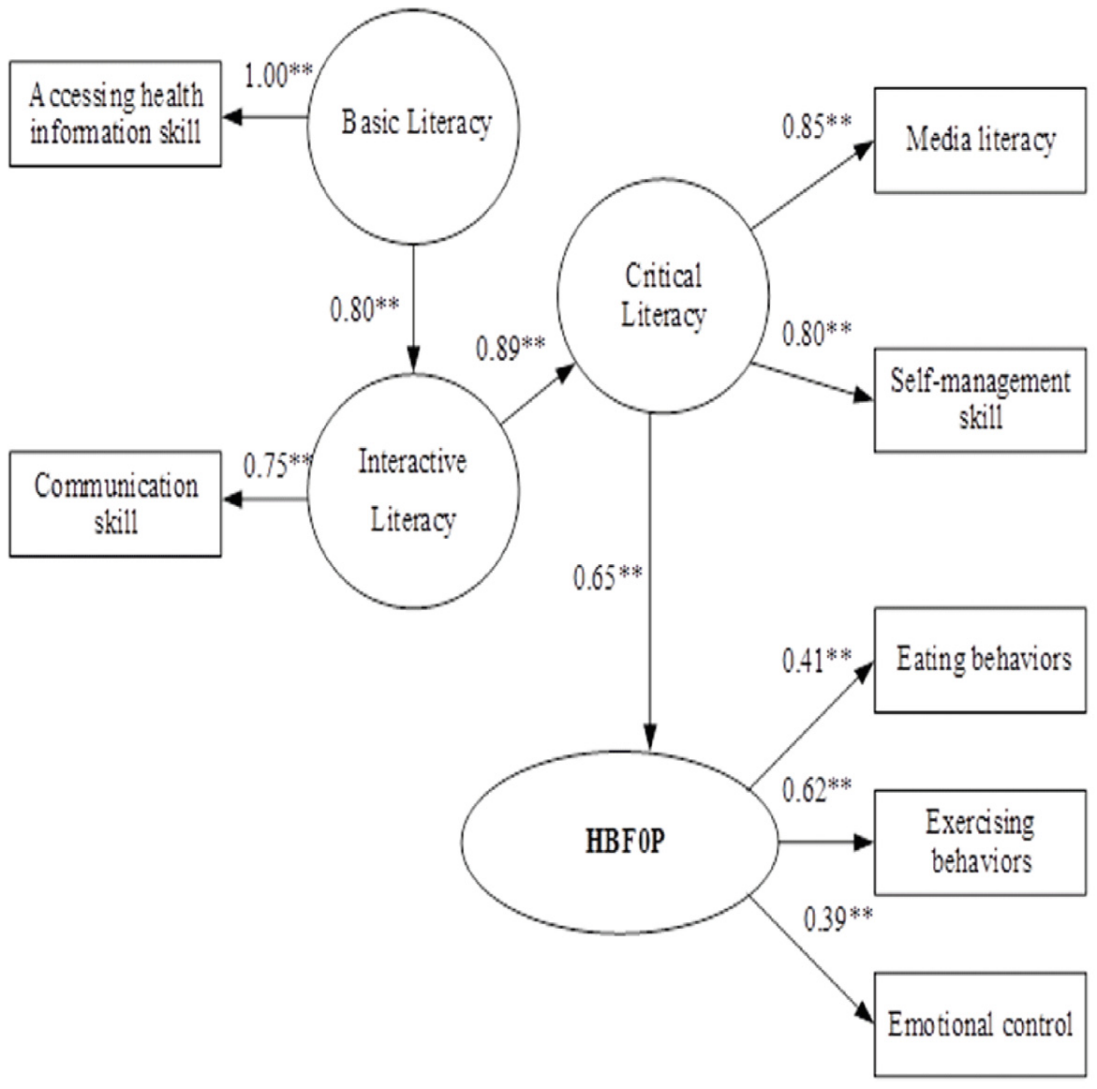
The causal model of health literacy on health behavior for obesity prevention among primary school students in Bangkok, Thailand. Chi-Square = 13.68, df = 7, P-value = 0.05721, RMSEA = 0.040, Standardized RMR = 0.017 NFI = 0.99 GFI = 0.99 AGFI = 0.97.

The causal model (CM) between HLFOP and HBFOP indicated that HLFOP had direct influence on HBFOP in Critical Literacy with effect size at 0.65, and indirect influence in Basic Literacy and Interactive Literacy through Critical Literacy, with effect sizes at 0.46. and 0.58, respectively (p< 0.01). For HLFOP, Critical Literacy was directly influenced by Interactive Literacy, with effect size at 0.89, and indirect influence from Basic Literacy through Interactive Literacy with effect size 0.71, (p <0.01). For HLFOP, Interactive Literacy was directly influenced by Basic Literacy with effect size at 0.80 (p < 0.01). Squared multiple correlation for SEM of HBFOP (R ^2^) = 0.21, which indicated that variables in the model could explain variances of HBFOP at 21% ( Table 2). The CM was consistent with the empirical data, as seen in Figure 4.

**Table 2.** Analysis of the influence of variables in the causal model of health literacy for obesity prevention (HLFOP) and health behavior for obesity prevention (HBFOP).

Exogenous variables	Basic Literacy (BL)			Interactive Literacy (IL)			Critical Literacy (CL)		
Endogenous variables	TE	IE	DE	TE	IE	DE	TE	IE	DE
IL	0.80 ^**^ (0.03)	-	0.80 ^**^ (0.03)	-	-	-	-	-	-
CL	0.71 ^**^ (0.03)	0.71 ^**^ (0.03)	-	0.89 ^**^ (0.08)	-	0.89 ^**^ (0.08)	-	-	-
HBFOP	0.46 ^**^ (0.02)	0.46 ^**^ (0.02)	- -	0.58 ^**^ (0.04)	0.58 ^**^ (0.04)	- -	0.65 ^**^ (0.03)	- -	0.65 ^**^ (0.03)
Chi-Square = 13.68, df = 7, P-value = 0.05721, RMSEA = 0.040, Standardized RMR = 0.017, NFI = 0.99, GFI = 0.99 Adjusted Goodness AGFI = 0.97									
Squared Multiple Correlation for Structural Equation of Endogenous Variables									
				IL		CL		HBFOP	
				R ^2^ = 0.63		R ^2^ = 0.50		R ^2^ = 0.21	
Reliabilities of Observable Variables									
		Accessing health information skill 1.00		Communication skill 0.57		Media literacy 0.73		Self- management skill 0.63	
Metrix of Variables Relationship									
		IL		CL		HBFOP		BL	
IL CL HBFOP BL		1.00 0.89 0.58 0.80		1.00 0.65 0.71		1.00 0.46		1.00	

** p< .01

## Discussion

The three factors of HLFOP included in this study, namely Basic Literacy (accessing health information skills), Interactive Literacy (communication skills), and Critical Literacy (ML and self-management skills), were consistent with the study by Tripetchsriurai and Kedcham who developed a HL scale for obesity among the secondary school students, grade 9 in Thailand, which found that HL consisted of self-management, accessing information and health care, communication for health promotion and reduction of risky health conditions, and media awareness ( Tripetchsriurai & Kedcham, 2017). Our HLFOB was also consistent with Nutbeam’s three factors: Basic/Functional Heath Literacy, Interactive Heath Literacy and Critical Heath Literacy ( Nutbeam, 2008), along with Manganello’s (2008) four levels of HL in adolescents (Functional, Interactive, Critical and ML). The three factors of HBFOP included in this study were Eating Behaviours, Exercising Behaviours and Emotional Control. These were consistent with the WHO (2000), “3E-2S” (Principles of NCD’s Prevention) of the Ministry of Public Health, Thailand ( Ministry of Public Health, 2016), and the Principles of Medical Practices and Treatment of Obesity Prevention in Thai Children ( Society of Pediatric Nutrition of Thailand, 2014).

For the HLFOP and HBFOP instrument development, the IOC values were between 0.8–1.0, which, as they are higher than 0.6 ( Hambleton, 1984), were found to be acceptable. In addition, the reliability values as calculated by Cronbach’s alpha were between 0.73–0.87, which higher is than the criteria for reliability of 0.7 ( Cronbach, 1990). The item correlation coefficient values were between 0.30–0.75, which is higher than the criteria of 0.2 ( Hambleton, 1984). The CFA indicated that factor loading of observable variables were statistically significant, mostly higher than 0.3 ( Hair *et al*., 2010). HLFOP contained factor loading between 0.69–0.87, whereas HBFOP was at 0.37–0.72. CFA of both scales were consistent with the empirical data. Therefore, it can be confirmed that these two scales were eligible to assess HLFOP and HBFOP in primary school students in the Bangkok Metropolis.

In the primary school students, HLFOP was primarily found to be at a low level (71.5%), with 26.5% of students showing a moderate level and 2% showing a high level. For HBFOP, most students had a moderate level (57.66%), with 39.17% at a low level and 3.17% at a high level. These findings were consistent with a previous study that indicated that HLFOP level of obese children aged 10–14 years were 60% with low levels, 38.4% with moderate, and 1.3% with high. In these children, 58.4% of children were found to have an overall HBFOP at moderate level ( Intarakamhang & Intarakamhang, 2017). Mean scores of the HLFOP in all sub-scales were also found at the low level: the lowest scores were mostly found in the communication skill (82.2%), self-management skill (60.33%), media literacy (58.2%), and accessing health information skill (52.67%), which indicated that most students still maintained low level of HLFOP in all sub-scales resulting in HBFOP at moderate and low levels. Therefore, HLFOP in all factors (Basic, Interactive and Critical Literacies) should be promoted to enhance both cognitive and social skills, which encourage individual’s motivation and competencies to be able to wisely access, understand and use various information sources and keep healthy ( Nutbeam, 2000; WHO, 2009b).

Since children and adolescents themselves have less access to healthcare services, and their development process needs more learning skills to grow to be healthy adults, information technology can be used to promote ML and health among this population. Similarly, they take in a huge amount of information through internet access, which can be used to provide a proactive approach. This allows them to get access to useful information about health and health services available, which can reduce expenses in healthcare services when they are older. Thus, HL development is supposed to be performed based on three aspects, including individual, interaction and society with a variety of developmental models ( Manganello, 2008).

This is especially true for low HL groups. There should be consideration of the needs and preferences of students or people with low HL when determining channels of health information dissemination. Implementing interventions should be considered to develop health information-seeking skills in the population and carefully prepare information and materials that are easily accessible and understandable ( Manganello *et al*., 2017 This is shown in a study of Hausmann *et al*. (2017) examining the use of social media in 204 children aged 12 years and above, attending a primary care adolescent and young adult clinic. The study showed that the adolescents and the young adults valued their privacy and the protection of their personal data. It was further found that 51.5% of the participants gave out their health information on social media, 48.5% did not give out and only 25% of them believed that social media could provide them with useful health information. Few of the participants connected with their health care team on social media, while most of them did not want to use this method; texting was preferred ( Hausmann *et al*., 2017).

Our findings also confirmed the causal relationship of HLFOP influence on HBFOP. This was shown through Basic Literacy (accessing health information skill) through Interactive Literacy (communication skill), with an effect size at 0.80, through Critical Literacy (ML and self-management skill), with effect size at 0.89, and through HBFOP with effect size 0.65. These results are consistent with a study by Chang, where HL in high and low groups were correlated with HB in nutrition ( Chang, 2011); children with higher HL were less likely to be obese and underweight. In addition, children who did not have regular physical activity, or have sugar-sweetened beverage intake are more likely to report being overweight or obese ( Shu-Fang *et al*., 2016). In addition, our results are consistent with a study that showed that exercising habits were positively related to self-management and ML, at 0.01 and 0.05, respectively ( Thipwong & Numphol, 2014). Our results were also consistent with the study of Intarakamhang & Intarakamhang (2017) in that HL influenced HB of obese school children, and basic literacy had an influence on HB through interactive literacy and critical literacy with effect sizes of 0.76, 0.97, and 0.55. The path analysis of HL model component revealed that HL started from health information and service access, directly passed through communication, media literacy to decision making with effect sizes of 0.63, 0.93, 0.98, and 0.05, respectively.

In our study, HLFOP had significant influences on HBFOP in terms of eating, exercises, and emotional control, resulting in better health conditions of the elementary school students. This is compatible with the theory of Behavior Modification based on Cognitive Behavior Theory in which 1) behaviors are affected by cognitive process; 2) cognitive process can be modified; 3) behavior can be modified by cognitive process changing ( Dobson, 2010). This is also consistent with Bandura’s Social Cognitive Theory ( Bandura, 1986) where individual behavior changes occur in a social context with a dynamic and reciprocal interaction of the person, environment and behavior. Research has shown that sport participation is related to feelings of social competence, and this relationship increases across late childhood into early adolescence. There are significant associations with sport at baseline and a significant association with sport over time on self-perceived social competence for both men and women ( Bedard *et al*., 2020). Moreover, Manganello asserted that HL would resulted in HB, reduction in health costs, health services use, and living healthy lifestyles as evaluated by exercise, eating, emotion, smoking, drinking and drug consumption. But to succeed, individuals must be supported by policies ( Manganello, 2008). This was confirmed with research showing that having strong or weak policies was significantly associated with lower BMI z-scores, lower odds of overweight or obesity, and better dietary outcomes, relative to no policy ( Manganello *et al*., 2017).

Therefore, we recommend that evaluation of HLFOP and HBFOP for primary school students should be firstly established in the National Policy and Principles of Health Development. Promotion strategies involving HL basic literacy in healthcare information should be managed in an easy way to access and understand. Social skills or interactive literacy for increasing channels of communication and learning should be practiced. ML and self-management should also be applied in the children’s daily lives.

### Implications and contributions

This research should be beneficial for teachers, instructors, paediatric nurses, school health nurses and related personnel in policy planning, and formulating activities to promote HLFOP and HBFOP. Curriculum development in this aspect should be constructed for primary students in their schools and community. Finally, further research in HL and HB should be conducted. Other factors related to the contexts of individuals, societies and appropriate policy for urbanization should be studied.

## Data availability

### Underlying data

Underlying data cannot be shared as the ethical committee that approved this study states that only aggregated data can be shared openly. In addition, the consent form that parents/children signed explicitly stated that the data resulting from the study would not be openly shared. Researchers interested in accessing the data will need to submit an official letter of request for the data to Navamindradhiraj University, and will be asked to confirm that they will not violate the ethical standards of the ethical committee and protect the anonymity of the participants. Researchers can contact the corresponding author, who can facilitate this process.

### Extended data

Open Science Framework: The causal model of health literacy and health behavior for obesity prevention among primary school students in Bangkok, Thailand, https://doi.org/10.17605/OSF.IO/YVA6Z ( Thongsong & Neranon, 2021).

This project contains the following extended data:

- HLFOP and HBFOP research instrument in English

Data are available under the terms of the Creative Commons Zero "No rights reserved" data waiver (CC0 1.0 Public domain dedication).
