# Supplementary material for: The causal model of health literacy and health behavior for obesity prevention among primary school students in Bangkok, Thailand
Source: F1000Res. 2021 Dec 21;9:1342. Originally published 2020 Nov 18. [Version 2] doi: 10.12688/f1000research.26249.2 (PMC8822475; doi:10.12688/f1000research.26249.2)
Supplement: Peer review history

# Peer review: Reviewer response for version 2

**DOI:** 10.5256/f1000research.79795.r116811

**Published:** 2022-01-18

**Authors:** Tuyen Van Duong

**Affiliations:** 1 School of Nutrition and Health Sciences, Taipei Medical University, Taipei, Taiwan

Dear Authors,

 Thanks for addressing my previous comments. I have no further ones. Looking forward to reading your completed version.

Is the work clearly and accurately presented and does it cite the current literature?

Yes

If applicable, is the statistical analysis and its interpretation appropriate?

Partly

Are all the source data underlying the results available to ensure full reproducibility?

Partly

Is the study design appropriate and is the work technically sound?

Yes

Are the conclusions drawn adequately supported by the results?

Yes

Are sufficient details of methods and analysis provided to allow replication by others?

Partly

Reviewer Expertise:

Health literacy, healthy diet literacy, health-related behaviors, physical activity, mental health, health-related quality of life

I confirm that I have read this submission and believe that I have an appropriate level of expertise to confirm that it is of an acceptable scientific standard.

# Peer review: Reviewer response for version 1

**DOI:** 10.5256/f1000research.28971.r89153

**Published:** 2021-08-03

**Authors:** Tuyen Van Duong

**Affiliations:** 1 School of Nutrition and Health Sciences, Taipei Medical University, Taipei, Taiwan

The authors conducted a cross-sectional study on 600 primary school students to examine the association between health literacy and health behaviors. Significant associations were found. The study was ethically conducted. The manuscript was well-developed. However, I have some suggestions and comments to improve the logic and quality of the manuscript as below.

1. This is a cross-sectional study, and you used the SEM model to test the association. I suggest not using the word “causal relationship”. The title and purpose should be revised accordingly.
2. The presentation of the Introduction, Methods, Results and Discussion should also be revised. The authors should refine the writing throughout the manuscript to avoid mentioning "causal relationship".
3. In Phase 1, please refer to some guidelines for the development and validation of a research tool, e.g. Boateng *et al*., 2018 ^1^.
4. It is better to have a chart for sampling.
5. For the sampling, how did the authors approach and collect the study participants in one school? Did you have one or two more steps to recruit students by classes, grades (or age) to have a well-represented sample?
6. Which social demographic information did you collect? Why don’t you adjust these in the regression model?
7. Sections two and three of the questionnaire consist of positive and negative items. Authors should explain clearly which items, how to code, and calculate the score. The subdomains/subscales should also be explained (e.g. how many items are in each domain).
8. More details are needed in the statistical section.
9. In Figure 1, Figure 2, and Figure 3, there should be observational variables and latent variables. However, the authors seem to calculate the scores for subscales (e.g. media literacy, eating behavior). These are not the raw observational variables. There should be 30 items for HLFOP, and 24 items for HBFOP. Please explain and rerun the SEM models. You can add the finding in the supplementary documents if necessary.

Is the work clearly and accurately presented and does it cite the current literature?

Yes

If applicable, is the statistical analysis and its interpretation appropriate?

Partly

Are all the source data underlying the results available to ensure full reproducibility?

Partly

Is the study design appropriate and is the work technically sound?

Yes

Are the conclusions drawn adequately supported by the results?

Yes

Are sufficient details of methods and analysis provided to allow replication by others?

Partly

Reviewer Expertise:

Health literacy, healthy diet literacy, health-related behaviors, physical activity, mental health, health-related quality of life

I confirm that I have read this submission and believe that I have an appropriate level of expertise to confirm that it is of an acceptable scientific standard, however I have significant reservations, as outlined above.

## References

[1] : Best Practices for Developing and Validating Scales for Health, Social, and Behavioral Research: A Primer. *Front Public Health* .2018;6: 10.3389/fpubh.2018.00149 149 10.3389/fpubh.2018.00149 29942800PMC6004510

# Response

**Published:** 2021-08-20

**Authors:** Ladaporn Thongsong

**Affiliations:** Department of Pediatric Nursing, Kuakarun Faculty of Nursing, Navamindradhiraj University, Thailand

Dear Reviewer,

 I am grateful for your guidance. Your guidance is very helpful in our study. We will be happy to answer your questions and modify the work according to your suggestions.

 About your questions:

 1. This is a cross-sectional study, and you used the SEM model to test the association. I suggest not using the word “causal relationship”. The title and purpose should be revised accordingly.

**Reply:** I agree to revise the title and purpose that you suggest.

 2. The presentation of the Introduction, Methods, Results and Discussion should also be revised. The authors should refine the writing throughout the manuscript to avoid mentioning "causal relationship".

**Reply:** I agree to revise what you suggest.

 3. In Phase 1, please refer to some guidelines for the development and validation of a research tool, e.g. Boateng *et al*., 2018 ^1^ .

**Reply: **Thank you very much for the good advice and good article. This research was development and validation of research tools according to the guideline of Six-Stage Process for Structural Equation Modeling (Hair JF, Black WC, Babin BJ, *et al.,* 2010) and many research, e.g. Zamir LD, Lemish D, Gofin R, 2011, Manganello JA, DeVellis RF, Davis TC, *et al.*, 2015 *,* Intarakamhang U, Intarakamhang P, 2017, Tripetchsriurai N, Kedcham D *,* 2017, and especially, following the study of Thai people, the Behavioral Science Research Institute (2014b) and the Department of Health Service Support (2016). However, I will follow the suggestion that refers to some guidelines for the development and validation of a research tool into the revised article.

 4. It is better to have a chart for sampling.

**Reply:** Described in Article 5.

 5. For the sampling, how did the authors approach and collect the study participants in one school? Did you have one or two more steps to recruit students by classes, grades (or age) to have a well-represented sample?

**Reply: **The 600 students were selected using multi-stage random sampling from all parts of Bangkok. For a more convenient selection for research participants, the researchers categorized all parts of Bangkok, generally comprised of 50 areas in total, into three main area categories, i.e. outer, central, and inner zones. Simple random sampling was then used to select two areas from each zone, contributing to the total of six areas, and one school in each area was further selected. Lastly, stratified random sampling was adopted to sample the target student participants from the three categorized zones, resulting in 194 students from the outer zone, 252 students from the central zone, and 154 students from the inner zone.

 Inclusion criteria: primary school students aged 9–13 years studying in grades 4–6 (or Prathomsuksa 4–6) in schools under the Office of Bangkok Metropolitan Administration (BMA), with the ability to read, write, and communicate with normal movement, without congenital disease of metabolic syndrome and not being in weight control programs.

 As a chart for sampling.



 6. Which social demographic information did you collect? Why don’t you adjust these in the regression model?

**Reply:** The aim of the study was to develop a research instrument to study the levels of health literacy for obesity prevention (HLFOP), as well as health behavior for obesity prevention (HBFOP), investigating the causal model between health literacy and health behavior for obesity prevention among primary school students in Bangkok, Thailand. Thus, the presentation of this research focuses on the causal model. However, that is a good suggestion for the new issue.

 7. Sections two and three of the questionnaire consist of positive and negative items. Authors should explain clearly which items, how to code, and calculate the score. The subdomains/subscales should also be explained (e.g. how many items are in each domain).

**Reply: **Thank you for the good advice. About the tool subdomains/subscales that has been verified. Some of them have already been explained in the results section. However, I will explain further as suggested in the revised article.

 8. More details are needed in the statistical section.

**Reply: **Described in Article 9

 9. In Figure 1, Figure 2, and Figure 3, there should be observational variables and latent variables. However, the authors seem to calculate the scores for subscales (e.g. media literacy, eating behavior). These are not the raw observational variables. There should be 30 items for HLFOP, and 24 items for HBFOP. Please explain and rerun the SEM models. You can add the finding in the supplementary documents if necessary.

**Reply:** Because the aim of the study was to develop a research instrument to study the levels of health literacy for obesity prevention (HLFOP), as well as health behavior for obesity prevention (HBFOP), investigated the causal model between health literacy and health behavior for obesity prevention among primary school students in Bangkok, Thailand.

 Therefore, it is expected that we will have quality tools based on research (HLFOP and HBFOP) that can be used for the assessment of Thai children in Bangkok.

 In the article, the development process and validity and reliability of the instrument are presented in phase 1 and after phase 2. In Phase 2, the researchers used quality tools obtained from Phase 1 to collect data with a sample of 600 students.

 After Phase 2, the tools were brought to check the quality again (the reliability of the instrument was analyzed using Cronbach's alpha and exploratory factor analysis (EFA) and confirmatory factor analysis (CFA) were utilized to analyze construct validity).

 Figure 1, Figure 2, Figure 3, reports only the results of the instruments that passed the quality check after phase 2.

 Results Development of the research instrument, HLFOP, the construct validity of 18 items (out of 30) were approved by EFA. Three categories were classified: Basic Literacy (4 items on accessing health information skills); Interactive Literacy (5 items on communication skills); and Critical Literacy (4 items on ML and 5 items on self-management skills). Cronbach’s alpha for each category was 0.87, 0.78, 0.84, 0.76 and 0.81, respectively. Item correlation coefficient was between 0.3–0.75. KMO = 0.928, Bartlett's Test = 3737.4 (p<0.000). Factor loading was between 0.69–0.87. The CFA was consistent with the empirical data (Chi-Square = 0.96, df =1, P-value = 0.33, RMSEA = 0.000, Standardized RMR = 0.0058, NFI = 1, Goodness of Fit Index (GFI) = 1, Adjusted Goodness of Fit Index (AGFI) = 0.99; Figure 1).

 Results Development of the research instrument, HBFOP, the construct validity of 20 items (out of 24) were approved by EFA. Three categories were classified: Eating Behaviours (12 items); Exercising Behaviours (4 items); and Emotional Control (4 items). Overall Cronbach’s alpha was 0.73. Item correlation coefficient was between 0.3–0.68. KMO = 0.863, Bartlett’s’ Test = 3412.40 (p<0.000). Factor loading was between 0.37–0.72. The CFA was found consistent with the empirical data (Chi-square = 0.00, df = 1, p-value = 0.977, RMSEA = 0.00; Figure 2).

 For Figure 3, a causal model of HLFOP on HBFOP.

 Best regards,

 Thongsong L. and Neranon W.

# Response

**Published:** 2021-11-25

**Authors:** wanida neranon

**Affiliations:** Navamindradhiraj University, Thailand

1. The title, purpose, and manuscript have been revised accordingly not using the word “causal relationship”.
2. Refers to some guidelines for the development and validation of a research tool into the revised article.
3. Add a chart for sampling. *Participants*. Figure 1.
4. Explain the structure of the study instrument, sections two and three of the questionnaires about positive and negative items. Amount of item and calculate the score.
5. The unmodified parts were old Figure 1, Figure 2, Figure 3, (new version of Figure 2, Figure 3, Figure 4) because of reports only on quality instruments that passed the quality check after phase 2, according to the research objectives to develop a research instrument to study the levels of HLFOP, as well as HBFOP.

# Peer review: Reviewer response for version 1

**DOI:** 10.5256/f1000research.28971.r75778

**Published:** 2021-02-24

**Authors:** Supamas Angsuchoti

**Affiliations:** 1 Sukhothai Thammathirat Open University, Nonthaburi, Thailand

The overview of the article is okay. But suggest to adjust slightly as follows:

1. According to this review (Hmosuwan & Ekphlakron), the last name translates into English, as spelled like this. “Homsuwan & Ekphlakron”. 
2. Page 5  In the topic Results, Development of the research instrument. From the EFA, it should be revised to CFA because the analysis results described was CFA. 
3. Page 5  item correlation, it should be revised to item total correlation. 
4. Page 6  The EFA, it should be revised to CFA. 
5. Page 6  observable factor, it should be revised to observable variables. 
6. Page 9 about effect size  0.9, it should be revised to 0.97. 

Is the work clearly and accurately presented and does it cite the current literature?

Yes

If applicable, is the statistical analysis and its interpretation appropriate?

Yes

Are all the source data underlying the results available to ensure full reproducibility?

Yes

Is the study design appropriate and is the work technically sound?

Yes

Are the conclusions drawn adequately supported by the results?

Yes

Are sufficient details of methods and analysis provided to allow replication by others?

Yes

Reviewer Expertise:

Statistics, Research and Evaluation in education

I confirm that I have read this submission and believe that I have an appropriate level of expertise to confirm that it is of an acceptable scientific standard.

# Response

**Published:** 2021-03-08

**Authors:** Ladaporn Thongsong

**Affiliations:** Department of Pediatric Nursing, Kuakarun Faculty of Nursing, Navamindradhiraj University, Thailand

Dear Reviewer

 I am grateful for your guidance. Your guidance is very helpful in our study. We will be happy to modify the work according to your suggestions.

 Best regards,

 Thongsong L and Neranon W

# Response

**Published:** 2021-05-31

**Authors:** wanida neranon

**Affiliations:** Navamindradhiraj University, Thailand

I agree with the suggestions. I will edit for suggestions.

# Response

**Published:** 2021-11-25

**Authors:** wanida neranon

**Affiliations:** Navamindradhiraj University, Thailand

Adjusted all points that have been suggested.
